# Association of G-quadruplex forming sequences with human mtDNA deletion breakpoints

**DOI:** 10.1186/1471-2164-15-677

**Published:** 2014-08-13

**Authors:** Dawei W Dong, Filipe Pereira, Steven P Barrett, Jill E Kolesar, Kajia Cao, Joana Damas, Liliya A Yatsunyk, F Brad Johnson, Brett A Kaufman

**Affiliations:** Department of Animal Biology, University of Pennsylvania School of Veterinary Medicine, Philadelphia, PA USA; Penn Genome Frontiers Institute, University of Pennsylvania, Philadelphia, PA USA; Institute of Molecular Pathology and Immunology, University of Porto, Porto, PORTUGAL; Interdisciplinary Centre of Marine and Environmental Research (CIIMAR/CIMAR), University of Porto, Porto, PORTUGAL; Department of Chemistry and Biochemistry, Swarthmore College, Swarthmore, PA USA; Department of Cell and Developmental Biology, University of Pennsylvania Perelman School of Medicine, Philadelphia, PA USA; Department of Pathology and Laboratory Medicine, University of Pennsylvania Perelman School of Medicine, Philadelphia, PA USA

**Keywords:** G-quadruplex, mtDNA deletions, Mitochondrial disease, Mitochondrial genome instability, Non-B DNA, Nucleic acid structures

## Abstract

**Background:**

Mitochondrial DNA (mtDNA) deletions cause disease and accumulate during aging, yet our understanding of the molecular mechanisms underlying their formation remains rudimentary. Guanine-quadruplex (GQ) DNA structures are associated with nuclear DNA instability in cancer; recent evidence indicates they can also form in mitochondrial nucleic acids, suggesting that these non-B DNA structures could be associated with mtDNA deletions. Currently, the multiple types of GQ sequences and their association with human mtDNA stability are unknown.

**Results:**

Here, we show an association between human mtDNA deletion breakpoint locations (sites where DNA ends rejoin after deletion of a section) and sequences with G-quadruplex forming potential (QFP), and establish the ability of selected sequences to form GQ *in vitro*. QFP contain four runs of either two or three consecutive guanines (2G and 3G, respectively), and we identified four types of QFP for subsequent analysis: intrastrand 2G, intrastrand 3G, duplex derived interstrand (ddi) 2G, and ddi 3G QFP sequences. We analyzed the position of each motif set relative to either 5*'* or 3*'* unique mtDNA deletion breakpoints, and found that intrastrand QFP sequences, but not ddi QFP sequences, showed significant association with mtDNA deletion breakpoint locations. Moreover, a large proportion of these QFP sequences occur at smaller distances to breakpoints relative to distribution-matched controls. The positive association of 2G QFP sequences persisted when breakpoints were divided into clinical subgroups. We tested *in vitro* GQ formation of representative mtDNA sequences containing these 2G QFP sequences and detected robust GQ structures by UV–VIS and CD spectroscopy. Notably, the most frequent deletion breakpoints, including those of the "common deletion", are bounded by 2G QFP sequence motifs.

**Conclusions:**

The potential for GQ to influence mitochondrial genome stability supports a high-priority investigation of these structures and their regulation in normal and pathological mitochondrial biology. These findings emphasize the potential importance of helicases that subsequently resolve GQ to maintain the stability of the mitochondrial genome.

**Electronic supplementary material:**

The online version of this article (doi:10.1186/1471-2164-15-677) contains supplementary material, which is available to authorized users.

## Background

The mitochondrial genome (mtDNA) is a multicopy DNA molecule that encodes essential components of the respiratory chain. A broad spectrum of human diseases [1, 2] stem from mtDNA mutations, which cause mitochondrial dysfunction and multisystem disorders [3, 4]. The most severe mtDNA mutations are deletions, which cause respiratory dysfunction at a lower mutation load than point mutations (reviewed in [1]). Based on epidemiological studies, single mtDNA deletions cause ~13-30% of primary mitochondrial disease [3, 5] and can arise from matrilineal transmission or sporadically during oogenesis. Multiple mtDNA deletions may also arise in a small proportion of patients afflicted with mitochondrial disease [6], and frequently occur due to mutation in one of several nuclear genes that influence mtDNA synthesis [7]. Multiple mtDNA deletions also occur in somatic tissue of all individuals and accumulate with age [8–11].

MtDNA deletion breakpoints show non-random distributions that presumably reflect the mechanisms underlying the formation of deletions. Several studies have attempted to identify sequence-related mechanisms for mtDNA deletion formation. The first sequence features identified were short direct repeats that contain or flank the deletion junction [12–14]. MtDNA deletion breakpoints can contain direct repeats of 3–13 bp, but these motifs are not universal features of deletions [14]. Direct repeats remain the most commonly hypothesized sequence-based mechanism for forming mtDNA deletions (ex. [15]), but leave unexplained a significant proportion of these mutations.

MtDNA contains numerous sequences with the potential to form non-B DNA structures, which may interfere with efficient DNA replication and repair. Supporting this notion, mtDNA fragments that contain deletion hot spots show altered electrophoretic mobility [16], possibly due to formation of stable non-B form secondary structures. Sequences predicted to form stable stem-loop or cruciform structures in single-stranded DNA overlap only a subset of the total documented 5′ and 3′ deletion breakpoints [17, 18], indicating the potential involvement of other non-B form structures in mtDNA instability.

Among non-B forms of DNA, G-quadruplex (GQ) structures exhibit characteristics that could implicate them in mitochondrial genome instability. GQs are stable secondary structures composed of two or more stacked planar guanine tetrads stabilized by cations such as potassium. These structures can occur in a single strand of DNA or RNA (intramolecular) or among two or more single-strands (intermolecular) [19]. A subset of intermolecular GQs has been speculated to form between runs of guanines derived from each strand of duplex DNA (duplex-derived intermolecular) [20]. In the nucleus, GQ structures can hinder DNA replication [21] and cause genome instability [21, 22], and sequences with quadruplex forming potential (QFP) have been associated with deletions and duplications in the genomic DNA of cancer cells [23]. Sequences with QFP are prevalent in yeast mtDNA [24] and the promoters of prokaryotic genes [25], but little is known about QFP sequences in the mammalian mitochondrial genome. In mammals, mitochondrial RNA containing the conserved sequence block 2 (CSB2) forms a GQ *in vitro* [26]. This GQ structure is predicted to affect the formation of a stable RNA primer [27, 28] used both in the formation of D-loop structures thought to associate mtDNA with the inner mitochondrial membrane [29, 30], and in priming first strand mtDNA replication initiation [31]. To date, only three-guanine (3G) QFP have been described in mtDNA [24, 26, 32].

The abundance of QFP sequences in yeast mtDNA [24] and potentially in mammals (this study), coupled with described effects of GQ on nuclear genome stability, make GQ structures of particular interest as potential agents in human mtDNA deletion formation. The potential for GQ structures to associate with mtDNA deletions has previously been suggested for a limited number of 3G QFP [32]. Here, we performed an examination of multiple categories of QFP sequences in human mtDNA. We analyzed enrichment across small distances between QFP and 5′ or 3′ breakpoints, and developed a reciprocal proximity method to analyze QFP and other sequence motifs for systematic association with 5′ and 3′ mtDNA breakpoints. Our results demonstrate that mtDNA QFP sequences show significant association with deletion breakpoints and are enriched with breakpoints over short distances at least as well as direct repeat sequences. All other structures tested failed to show such association. Not previously reported, the two-guanine (2G) QFP motif set is the only QFP motif that is systematically associated with both 5′ and 3′ breakpoints. We further show the capacity of selected 2G QFP to form GQ structures *in vitro*.

## Methods

### Identification of QFP sequences

**Figure 1:**
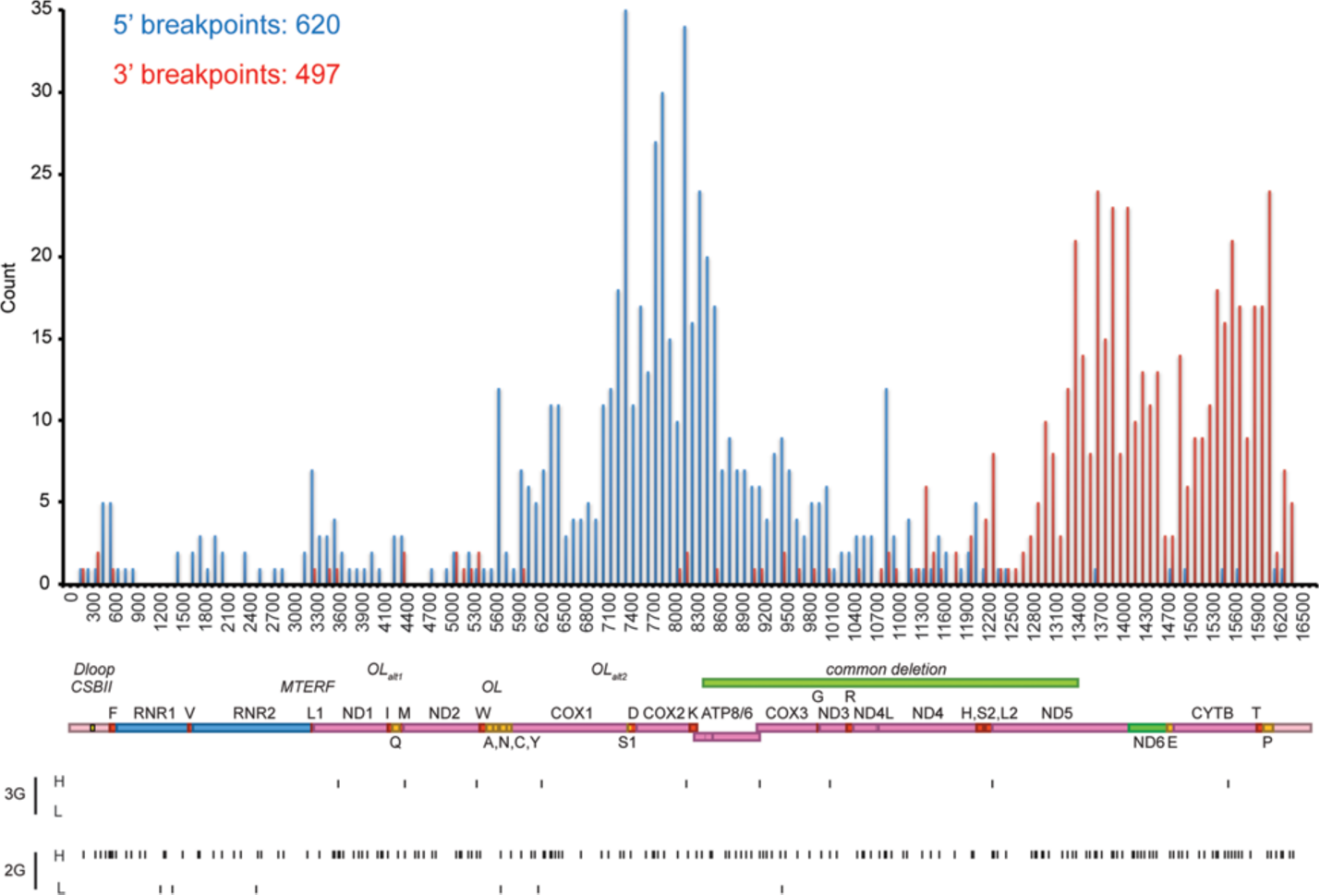
**Distribution and position of non-redundant 5′ and 3′ breakpoints in 730 unique mtDNA deletions and the occurrence of sequences with G-quadruplex forming potential (QFP).** 620 5*'* and 497 3*'* breakpoint sites are shown in the frequency histogram using 100 bp bins. The approximate positions of mitochondrially-encoded genes are shown below the histogram. 3G and 2G QFP sequence positions are shown below the mitochondrial genome map, with QFP located on heavy (H) or light (L) strands as indicated.

The intrastrand QFP sequences in the heavy and light strands of the human mitochondrial genome (NC_012920) were extracted using our own QFP predicting program (available upon request). This program analyzed the genome as a circular sequence. We selected a QFP length of 33 to allow the analysis of the maximum number of non-overlapping QFP clusters in the mitochondrial genome. The parameters were 33 nt maximum motif length, loop size 1 or larger, and minimum length of G-runs at two or three (2G or 3G QFP, respectively). In cases where overlapping QFP elements were found, only one element was chosen; to select the QFP element most likely to form a stable GQ, we used two methods based on previous studies: select the QFP with shorter length, and select the QFP with greater numbers of G tetrads [33–36]. The results are similar to the online QGRS Mapper [36]. Lists of midpoint positions for each QFP set were made by identifying the middle position of the QFP sequence, or by using the median of midpoints of remaining overlapping QFPs (i.e. overlapping QFP sequences with same length and same number of G tetrads; illustrated in Additional file 1: Figure S1). Midpoints of intrastrand 3G and 2G QFP are shown in Figure 1. Duplex-derived interstrand QFP (ddi QFP) sequences were identified by locating four G-runs where each run could be on either strand of mtDNA within a 33 nt analysis window, with intrastrand QFP sequences removed to avoid overlap between intra- and interstrand sets [20]. These would include GG-CC-GG-CC, GG-CC-CC-GG, and GG-GG-CC-CC, among multiple other arrangements.

### Identification of stem-loop/cruciform structures and repeat sequences

The prediction of stem-loop, cruciform and other hairpin-containing features across human mtDNA was carried out using the hybrid-ss-min core program of the UNAFold 3.8 software package [37]. For the sake of simplicity, the abbreviation SC will be used to define any of these different structural elements. The folding of single-stranded DNA was simulated at 37°C in 1 M sodium and no magnesium, using 100 nucleotides as the maximum distance between paired bases in each structure. The circular nature of the mtDNA was considered in the folding prediction. Python scripts were created for the identification of direct repeats (e.g., ATC-ATC), inverted repeats (e.g., ATC-CTA), complementary repeats (e.g., ATC-TAG) and inverted complementary repeats (e.g., ATC-GAT) in human mtDNA. Midpoints were used in all calculations. The circular representation of mtDNA was made using the Circos software package, version 0.62 [38].

### Minimal Distance Analysis (MDA)

**Figure 2:**
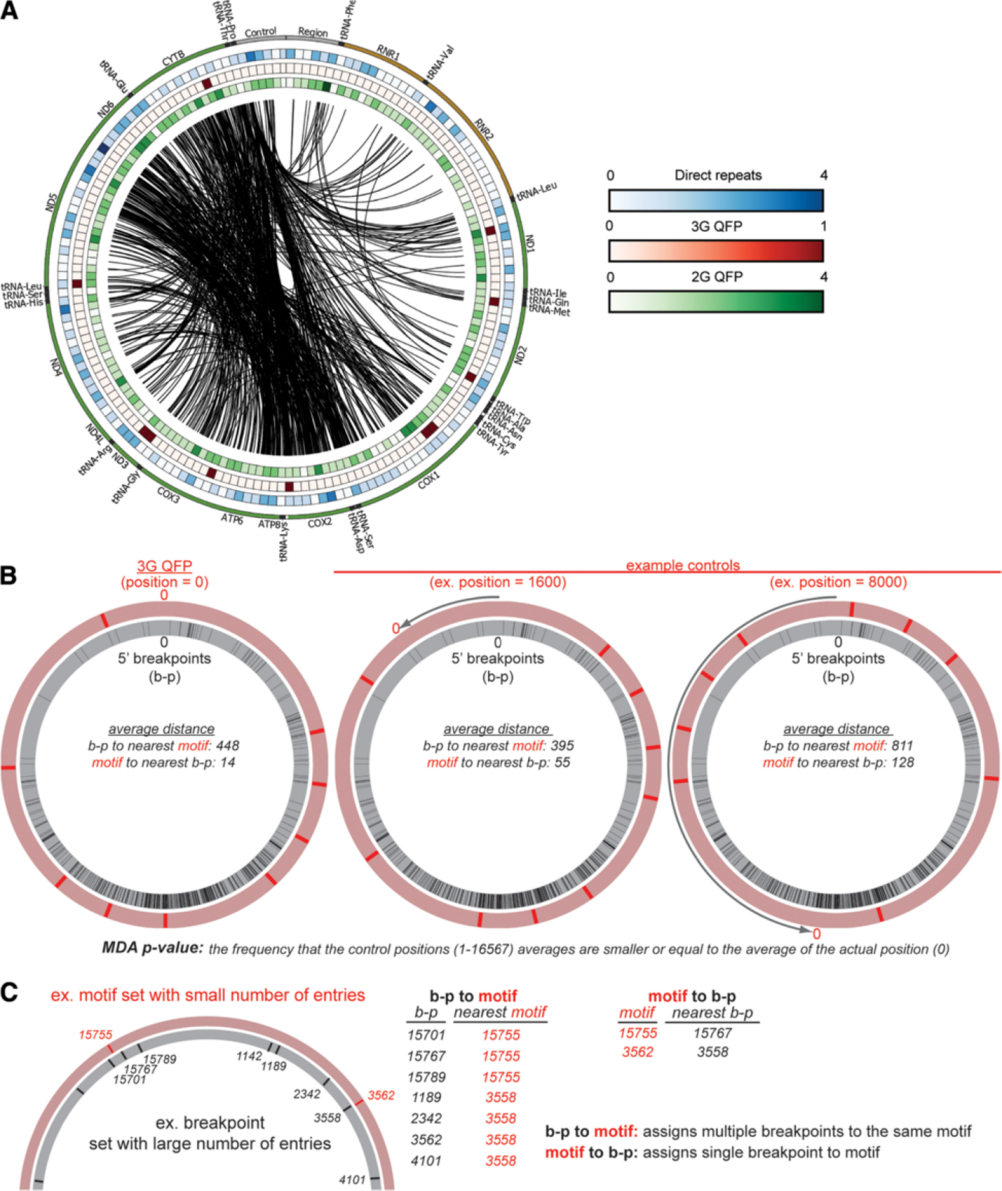
**Position of mtDNA deletion breakpoints relative to 2G QFP, 3G QFP, and direct repeat sequences. A)** Circular plot of the mitochondrial genome specifying the location of deletion breakpoints and structural motifs. The center black lines connect the 5*'* and 3*'* breakpoints for each of the 730 deletions. The outer track depicts the mitochondrial genome with annotated tRNA (black), rRNA (brown) and protein-coding (green) genes. The control region is highlighted in gray. The inner tracks indicate the number of direct repeats (blue/white gradient), 3G QFP motifs (red/white gradient) and 2G QFP (green/white gradient), all measured in 100-nt windows. If a motif overlapped two 100-nt windows, it was considered to be in both windows. **B)** Schematic demonstration of the spatial arrangement of 3G QFP sequences and 3’ breakpoints for actual position (left) and a rotation-generated control (right). **C)** Schematic demonstrating a limited data set illustrating breakpoints (b-p) and rare motifs. Because breakpoints outnumber motifs, b-p to motif calculations are interpreted with caution due to assignment of multiple breakpoints to the same motif. Association using MDA can occur using b-p to motif *or* motif to b-p, but need not necessarily occur in both.

The significance of association between structural motifs and either 5′ or 3′ mtDNA deletion breakpoints was determined by minimal distance analysis (MDA). Rather than simply asking if the number of events where the number of breakpoints with minimal distances smaller than a given value was significantly larger than chance for a given set of motifs (this was done by testing individual enrichment, explained in the next section), we wanted to know if a set of breakpoints as a whole (a "breakpoint set") was in proximity to a set of motifs (a "motif set"). To determine the significance of the proximity, we tabulated the average minimal distance for each member of a breakpoint set (all 5′ or all 3′ are considered separate sets) to its closest neighbor that is a member of a motif set and compared this actual value to that of control sets. Control sets were generated by rotating the motif set around the circular genome in 1-nucleotide (nt) increments relative to the breakpoint set. These control sets allow the determination of probability (p-value) that a breakpoint set is close to a motif set (b-p to motif). It is important to understand that the average minimal distance from a motif set to a breakpoint set is by definition not the same as the average minimal distance from the breakpoint set to the motif set. The reciprocal analysis (motif to b-p) determines the probability that the set of motifs are significantly close to the set of breakpoints. The difference between b-p to motif and motif to b-p is illustrated in Figure 2. The motif to b-p MDA is important for motif sets with a low number of entries (such as 3G QFP sequences) because such a small number of motifs cannot be near every breakpoint. Therefore, even in cases that do not show a significant association by b-p to motif MDA, there may be a subset of breakpoints closely associated with the rare motifs, which would be significant in motif to b-p MDA. The p-value in MDA was calculated as the fraction of controls that had average minimal distances smaller than or equal to the value determined for a given sequence motif set.

### Individual enrichment analysis

As in MDA, we used the minimal distance between breakpoints and a motif set to determine enrichment for individual motifs; however, here we are asking if the number (K) of the breakpoints with distances smaller than or equal to a given value (D) was significantly larger than chance. The chance probability (P) is calculated from rotational controls, i.e., for all possible position of a breakpoint relative to a motif set, the portion of b-p to motif distances smaller than or equal to the given value D. Note that this is different from MDA. In MDA, the b-p to motif distances is averaged over the b-p set. But here, individual distance is counted without averaging. For a total number of N breakpoints, the p-value is simply *1 - F(K;N,P)*, in which F(K;N,P) is the binomial.


Thus, a small p-value signifies an enrichment. Similarly, we can evaluate the enrichment of the number of structural motifs at close distances to mtDNA deletion breakpoints.

### Enrichment of motifs with both mtDNA deletion breakpoints

Simultaneously for both breakpoints, mtDNA deletions could be associated with 2G QFP or direct repeat sequences. To test this, the distance of the nearest 2G QFP sequence to each end of a deletion is measured. If both of the distances are less than or equal to 10 nt, the deletion is considered to be close to 2G QFP motifs. The actual number of deletions close to 2G QFP motifs is compared to the control, which is calculated by rotating the 2G QFP set relative to the deletion set for all controls. The p-value is calculated from binomial distribution, as is the case for single-end enrichment. For direct repeat sequences, this is performed by two types of calculations. The first type is identical to that done for 2G QFP sequences, by considering all direct repeat sequences as equivalent entries. For the second type, each deletion is compared with each direct repeat pair. The distance from the 5′ end of the deletion to the 5′ end of the direct repeat pair, and the distance from the 3′ end of the same deletion to the 3′ end of the same direct repeat pair, are calculated. If both distances are less than or equal to 10 nt, the deletion is considered to be close to the direct repeat pair.

### Spectroscopic studies

All reagents were molecular biology grade or better from Sigma (Sigma-Aldrich, St. Louis, MO) and oligonucleotides were synthesized by IDT (Coralville, IA) with standard purification. All spectroscopic analyses were performed as previously reported [39, 40]. In brief, ~5.0 μM DNA samples were annealed by heating to 95°C for 5 min followed by slow cooling to room temperature. Thermal difference spectra (TDS) were collected on a Cary 300 Varian Spectrophotometer equipped with a Peltier-thermostated cuvette holder. Circular dichroism (CD), thermal melting and wavelength scans were performed on an AVIV 410 spectropolarimeter equipped with a Peltier heating unit. While monitoring the signal at 264 nm, DNA samples were heated from 5 to 95°C at a rate of 10°C/min with an equilibration time of 1 min, an averaging time of 10 s, and a bandwidth of 1 nm in 10 mM lithium cacodylate buffer, pH 7.2, supplemented with 150 mM KCl. The data were analyzed using a two-state Van’t Hoff equation assuming constant enthalpy, ΔH. CD wavelength scans were collected on samples both before and after melting. Three scans, over a wavelength range of 220 to 350 nm, were recorded at 4°C with a bandwidth of 1 nm, an averaging time of 1 s, and a wavelength step of 1 nm. These scans were averaged, baseline corrected, smoothed and converted to molar ellipticity. All data analysis was done in Origin 8.1 (OriginLab, Northampton, MA).

## Results

### Identification of four types of QFP sequences in human mtDNA

We began our investigation by generating a list of human mtDNA sequences with the potential to form G-quadruplexes in single-stranded (intrastrand) or double-stranded (duplex-derived interstrand, or ddi) DNA. Algorithms designed to predict QFP sequences have been developed for both single-stranded (reviewed in [41]) and double-stranded sequences [20]. The two mtDNA strands have asymmetric nucleotide distribution, with adenine and guanine purines enriched on the heavy strand. As described in Methods, we identified 2G and 3G ddi QFP sequences, and intrastrand 2G QFP and 3G QFP sequences on the heavy strand of human mtDNA. We found only 2G QFP on the light strand due to the scarcity of guanine residues there. Strikingly, the number of QFP sequences in human mtDNA was significantly higher than the number found in random genomes with the identical nucleotide content and asymmetry (not shown). The positions of the non-overlapping intrastrand QFP sequences are shown in Figure 2 relative to a reference map for human mtDNA (NC_012920).

### 2G QFP and 3G QFP motifs, but not ddi QFP, are associated with human mtDNA deletion breakpoints

We next tested the null hypothesis that 5′ or 3′ mtDNA deletion breakpoints (b-p) were randomly positioned relative to QFP sequence sets (motif). We selected a set of 730 human mtDNA deletions that included associated clinical data [41], and identified all unique 5′ and 3′ deletion breakpoints in this collection (Figure 1). The positional enrichment of 3G QFP and 2G QFP sequences relative to unique mtDNA deletion breakpoints is shown in Figure 2A. Because QFP sequences could lie inside or outside the deleted sequence, we calculated the minimal distance in either direction from each breakpoint in a set (i.e. 5′ or 3′ b-p set) to the nearest QFP sequence midpoint (b-p to motif). To establish distribution-matched controls, the midpoint positions for a QFP sequence set were rotated around the circular mtDNA genome in 1 nt increments to generate 16,568 control data sets. In this minimal distance analysis (MDA), p-values were calculated by determining the frequency at which a control set’s average minimal distance from b-p to motif was smaller than or equal to the value for the actual data set (examples shown in Figure 2B,C). As such, an MDA p-value of 0.001 (b-p to motif) indicates that the average distance from a breakpoint to nearest QFP sequence motif was smaller than controls for all but 16 positions in the 16 kb genome. Furthermore, our controls are more restrictive than other randomized control sets [32], where changes in randomized distribution of motifs or breakpoints is sufficient to reject the null hypothesis.

The MDA results for intramolecular QFP motifs are shown in Table 1. To analyze motif sets with few entries, such as 3G QFP motifs, we performed motif to b-p MDA analysis to prevent false negative associations over long distances (explained in Figure 2B,C). We found that the distance from 3G QFP sequences to 5′ b-p and 3′ b-p were significantly smaller than controls via MDA (motif to b-p: p = 0.0037 and p = 0.020, respectively; Table 1, top box). Notably, both b-p to motif and motif to b-p MDA analyses achieved significance for 2G QFP and 3′ breakpoints (p = 0.0012 and p = 0.032, respectively), indicating that on average, not only are the 3′ b-p close to 2G QFPs, but also that 2G QFPs are close to 3′ b-ps. This association in both directions did not occur for any other motif set (Table 1). 2G and 3G ddi-QFP sequence motifs were also examined, even though the capacity for such motifs to form GQs is quite speculative [20]; both of these motifs failed to significantly associate with the 5′ or 3′ breakpoint sets in either type of MDA (Additional file 2: Table S1: b-p to motif; motif to b-p) and were not pursued further.

**Table 1:** **Quadruplex forming potential (QFP) and other structural motifs analyzed for minimum distance relationship to 5’ or 3’ human mtDNA deletion breakpoints**

		*Actual/control average distance*					
		5′ breakpoints(620)		3′ breakpoints(497)			
Motif	#	*b-p to motif*	*Motif to b-p*	*b-p to motif*	*Motif to b-p*	*Length(nt)*	*Example*
3G QFP	9	447/667	14/74***	742/667	48/224*	<33	GGG-GGG-GGG-GGG
2G QFP	178	26/36***	79/74	23/36**	186/224*	<33	GG-GG-GG-GG
SC structures	405	15/14	76/74	14/14	262/224	<100	
direct rpts.	137	44/63*	80/74	42/63*	200/224	11	ATC-ATC
inverted comp. rpts.	56	98/113	58/74	120/113	287/224	11	ATC-GAT
complementary rpts.	35	210/248	61/74	316/248	314/224	11	ATC-TAG
inverted rpts.	133	51/58	87/74	44/58	205/224	11	ATC-CTA

3G QFP and 2G QFP analyzed for breakpoints (b-p) to motif association and motif to b-p association for both 5′ and 3′ breakpoints. Actual and control distances are provided (rounded to the nearest integer). SC structure and sequence repeat predictions. At right, dashes in examples represent loops or intervening sequence. The frequency at which the control data showed a shorter average distance was tabulated to determine the p-value (* < 0.05; ** < 0.01, *** < 0.001; exact values shown in Additional file 2: Table S1). Statistically significant associations are indicated in bold.

### Direct repeat motifs, but not other DNA motifs, are associated with human mtDNA breakpoints

In addition to QFP motifs, we investigated the association of other DNA motifs with deletions by performing MDA on several types of sequence features. Stem-loop or cruciform (SC) structures predicted by the UNAFold algorithm are thought to associate with a limited number of the most common mtDNA deletion breakpoints [18]. Using UNAFold, we generated a list of predicted SC sequences and found that these sequences occur frequently (Table 1). However, MDA showed no association of SC structures with either 5′ or 3′ breakpoints. We conclude that these sequences identified by UNAFold, treated equally, do not explain the diverse population of deletion breakpoints, which is consistent with findings of others [15]. SC sequences have been shown, however, to strongly associate with specific deletion breakpoints among the most common deletions [17, 18], which supports the notion that multiple mechanisms may contribute to mtDNA deletion formation.

Additional DNA sequence motifs have been suggested to contribute to mtDNA instability. Recently, association studies have suggested a negative correlation for inverted complementary repeat sequences in mtDNA in maximum life span across species, possibly through increased deletion mutagenesis [42]. Furthermore, direct repeats of 11–15 bp have been frequently suggested to contribute to deletion breakpoint sites [12–14, 17, 43]. Direct repeat sequence pairs, inverted complementary repeat pairs, and individual stem-loop/cruciform structures are visualized (Additional file 3: Figure S2) to illustrate potential associations of these sequences with both 5′ and 3′ breakpoints in mtDNA deletions.

We next tested direct and inverted complementary repeat sets for association with mtDNA deletion breakpoints, followed by complementary and inverted repeats, which are not expected to basepair elsewhere in the genome. We selected an 11 nt repeat length because it was long enough to limit the number of triplet repeats but short enough to give the largest number of repeats within the range of perfect repeats associated with disease [12–14, 17, 43]. For comparison by MDA, each component of a pair was analyzed as a separate unit. This repeat pair uncoupling allowed us to test the null hypothesis that sequences capable of basepairing elsewhere in the genome are unassociated with 5′ or 3′ deletion breakpoints. We tested this hypothesis with b-p to motif and motif to b-p MDA (differences between MDA are illustrated in Figure 1B,C) and found significant association between direct repeat sequences and both 5′ and 3′ breakpoints in the b-p to motif MDA (Table 1). Although the p-values (p = 0.04, 0.03 for 5′ and 3′, respectively) are not as small as those for 2G QFP, we do believe that the association is significant, given that the p-values are much smaller in the enrichment studies when 5′ and 3′ ends are considered individually (see next section) and simultaneously (see Discussion). Inverted complementary repeats showed no significant breakpoint association, nor did complementary and inverted repeat sequences. The location of 2G QFP, 3G QFP, and direct repeat sequences relative to paired breakpoints in the mtDNA deletion set are shown in Figure 2A. The complete table of all p-values for all motifs and repeats is found in Additional file 2: Table S1. It should be noted that the p-values represent the significance of enrichment between motif and breakpoint relative to control data sets, but they do not allow qualitative comparison of two motifs with statistically significant enrichment.

### Human mtDNA deletion breakpoints are enriched near 2G QFP, 3G QFP, and direct repeat motifs

**Figure 3:**
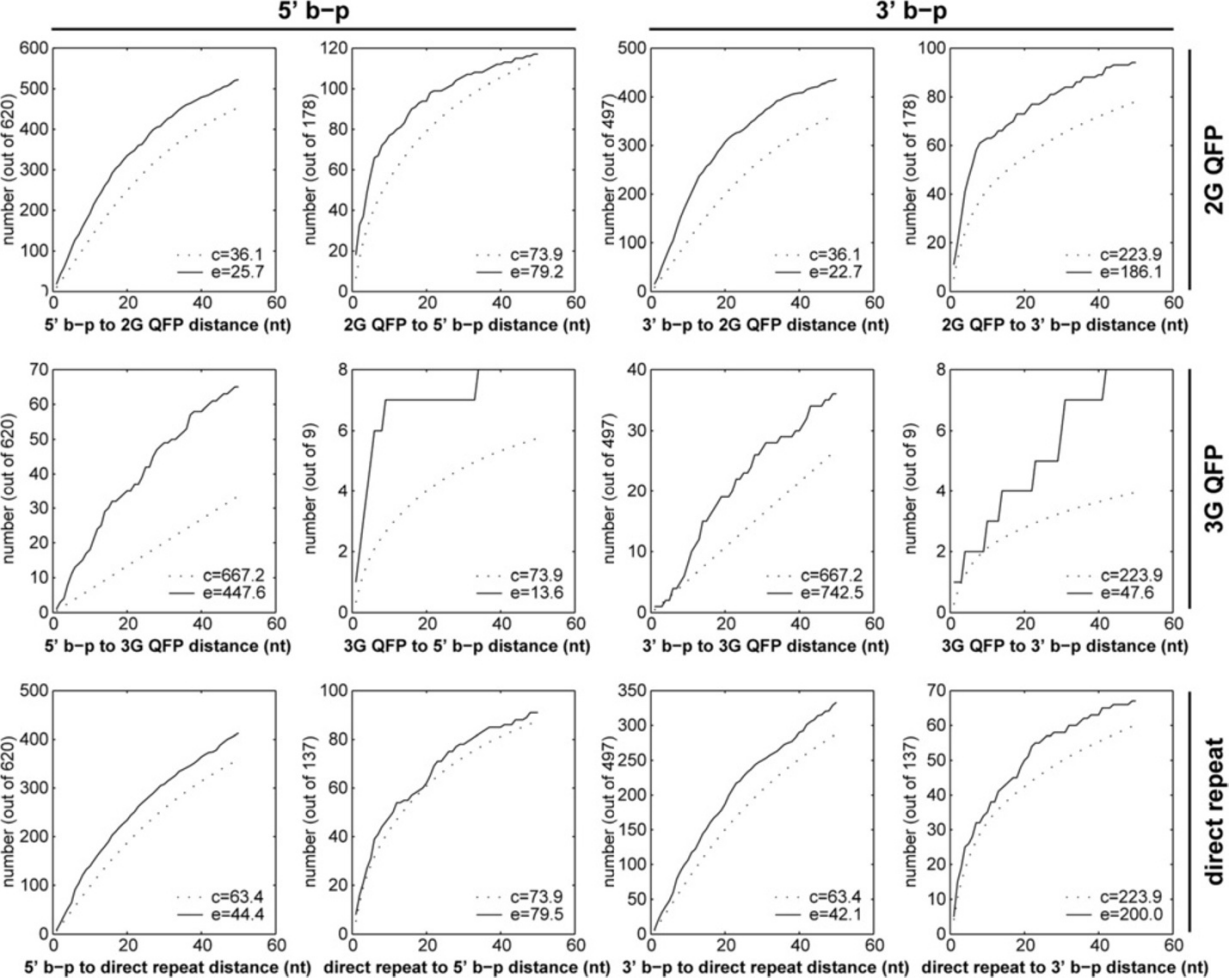
**Cumulative distribution of distances between 5′ or 3′ breakpoints and midpoints of 3G QFP, 2G QFP, and direct repeat sequences.** At each x-axis value, the y value is the number of experimental (minimum) distance ≤ x (solid line) or the average number of control (minimum) distance ≤ x (dashed line). The average minimal distance for control (c) and for experimental (e) are shown in the figure legend. Binomial p-values at 10 nt increments are provided in Additional file 4: Table S2.

We then examined the enrichment of individual 2G QFP, 3G QFP, and direct repeat sequences with 5′ or 3′ breakpoints (Figure 3). This contrasts to the minimal distance analysis, which considers the optimal positions between entire motif and breakpoint sets. Like the MDA, the enrichment between motifs and breakpoints can be visualized from the perspective of motifs (motif to b-p) or the perspective of breakpoints (b-p to motif). To that end, we plotted the number of breakpoints as a function of minimum distance to nearest motif (b-p to motifs) or the number of motifs as a function of the minimum distance to nearest breakpoint (motif to b-p), relative to their respective control values. Because a motif is most likely to exert an effect over a short distance, we show the proximal 50 nt on the x-axis in Figure 3. The average of all experimental values (i.e. not limited to proximal 50 nt) is shown alongside the average of all control data in each plot. A distance of less than 16 nt for QFP and 6 nt for direct repeat sequences potentially reflects overlap of the motif and breakpoint. 2G QFP and 3G QFP sequences show significant enrichment with both 5′ and 3′ breakpoints for in the vast majority of tests up to 50 nt (including both motif to b-p and b-p to motif analysis; binomial p-values shown in Additional file 4: Table S2). Direct repeat sequences demonstrate significant enrichment with 5′ and 3′ breakpoints in b-p to motif analysis, but show no significance at any distance in the motif to b-p analysis.

### Association of 2G QFP with the "common deletion"

**Figure 4:**
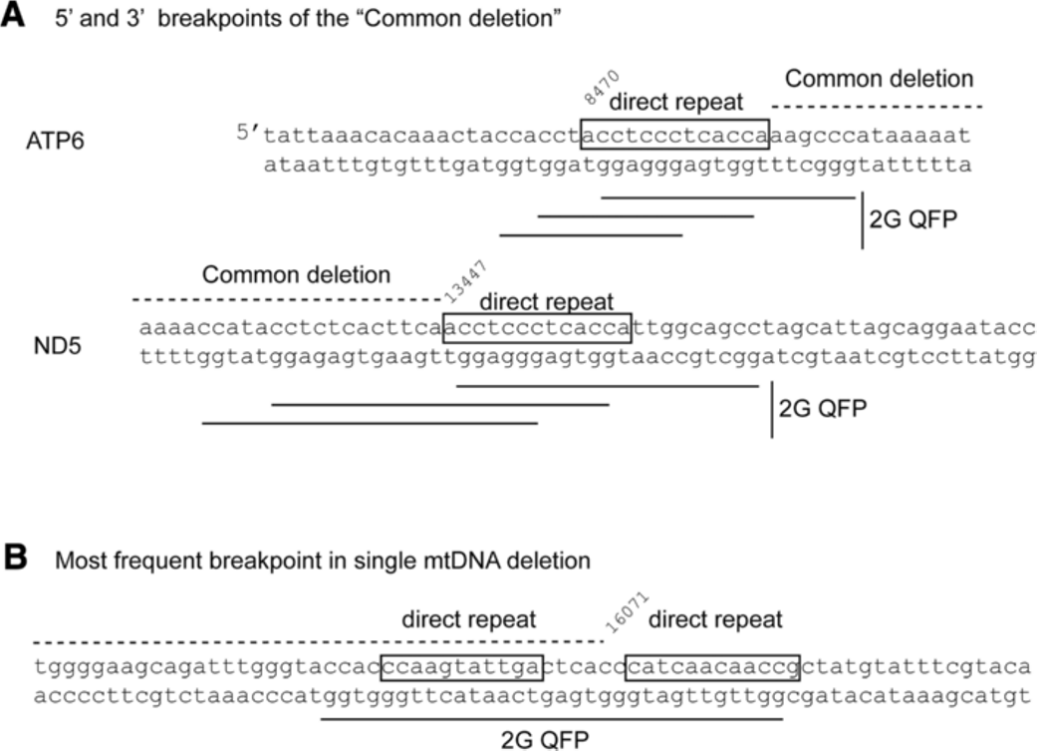
**Common deletion breakpoints contain direct repeats and 2G QFP sequences. A)** The "common deletion" breakpoints contain overlapping 2G QFP sequences. Boxes indicate the 13 nt direct repeats that comprise the breakpoint junction between ATP6 and ND5 in the common deletion. Direct repeat and flanking sequences in ATP6 and ND5 contain three overlapping QFP sequences. The N1b mtDNA sequence variant is 8472C > T. **B)** The highest frequency breakpoint is a 3’ breakpoint at 16071, which is right in the middle of a 2G QFP sequence. This region contains two different 12 nt direct repeat sequences, starting at 15990 or 16073. The cognate sites start at 1889 and 8637, respectively. Deletions involving 1889 or 8637 with the 16071 region are present in the deletion collection; however, there are approximately 200 unique deletions involving the 16071 region as the 3’ breakpoint for many other 5’ breakpoints [45].

We also found compelling evidence for association of 2G QFP with breakpoints of the "common deletion" [12]. We find three overlapping 2G QFP in the 13 bp repeats that mark the breakpoints of this deletion (Figure 4A). Importantly, the mtDNA sequence variant present in the N1b haplogroup (8472C > T) abolishes the guanine run shared by all three 2G QFP sequences at the 5’ breakpoint of the common deletion, and would be expected to prevent the formation of GQ structures at that position. In fact, the formation of the common deletion is significantly reduced by this mutation, which does not affect the distribution of other mtDNA deletions in this haplogroup [44], suggesting that the reference sequence must be intact for the common deletion to occur. Another 2G QFP sequence is present in a deletion breakpoint hot spot at nt 16071, which is the most common 3’ breakpoint, as it occurs in numerous deletions with various 5’ breakpoints [17, 45]. Here, a 2G QFP sequence overlaps with two different 12 nt direct repeat sequences (Figure 4B). This same region has been predicted to form stem-loop structures [17, 18]. Some precedent supports the connection of 2G QFP sequences with deletions, as previous reports analyzing regions of genome instability found degenerate 13-mer sequences that contain three 2G runs that associate with nuclear and mitochondrial deletions [46].

### Association of motifs with clinical categories of human mtDNA deletions

To establish how motifs associate with mtDNA deletions from specific clinical categories, we examined all QFP and repeat sequences for association with seven groups of recently described mtDNA deletions [45]. The so-called "common deletion" was the only deletion found in all seven groups. For each category, the distribution of 5’ and 3’ deletion breakpoints across mtDNA is shown by frequency histogram in Additional file 5: Figure S3. By MDA, 2G QFP sequences showed significant association with the 3′ breakpoints of the majority of clinical categories (six of seven), while 5’ breakpoints were associated with 2G QFP sequences in two of the categories (Additional file 6: Table S3A,B). Both 3G ddi QFP and 2G ddi QFP sequences showed association with individual categories, but never with both 5′ and 3′ breakpoints in the same category. Direct repeat sequences showed significant association with 3′ breakpoints from four of seven groups, but only one category of 5′ breakpoints. SC sequences and other control sequences failed to show broad association with any individual clinical category of deletions (Additional file 2: Table S1).

We noted that 2G QFP sequences showed nearly significant association with 5′ breakpoints in patients with single mtDNA deletions. To better understand this, we identified disease-specific mtDNA deletions from within this clinical group and determined the MDA values for 2G QFP sequence association with 5′ and 3′ breakpoints (Additional file 6: Table S3C,D). The distribution of mtDNA deletion breakpoints for Kearns-Sayre syndrome (KSS), progressive external opthalmoplegia (PEO), and Pearson’s syndrome (PS) is shown in a frequency histogram in Additional file 7: Figure S4. MDA showed significant association of 2G QFP sequences with KSS and PEO deletion breakpoints but not those found in PS. Comparisons of mtDNA deletion locations among these populations showed limited overlap between PS and both KSS and PEO sets, but extensive overlap between KSS and PEO sets (Additional file 8: Figure S5). These results suggest that 2G QFP sequences are in close association with at least a subset of single mtDNA deletion breakpoints.

### 2G QFP sequences form *bona fide*G-quadruplexes *in vitro*

**Figure 5:**
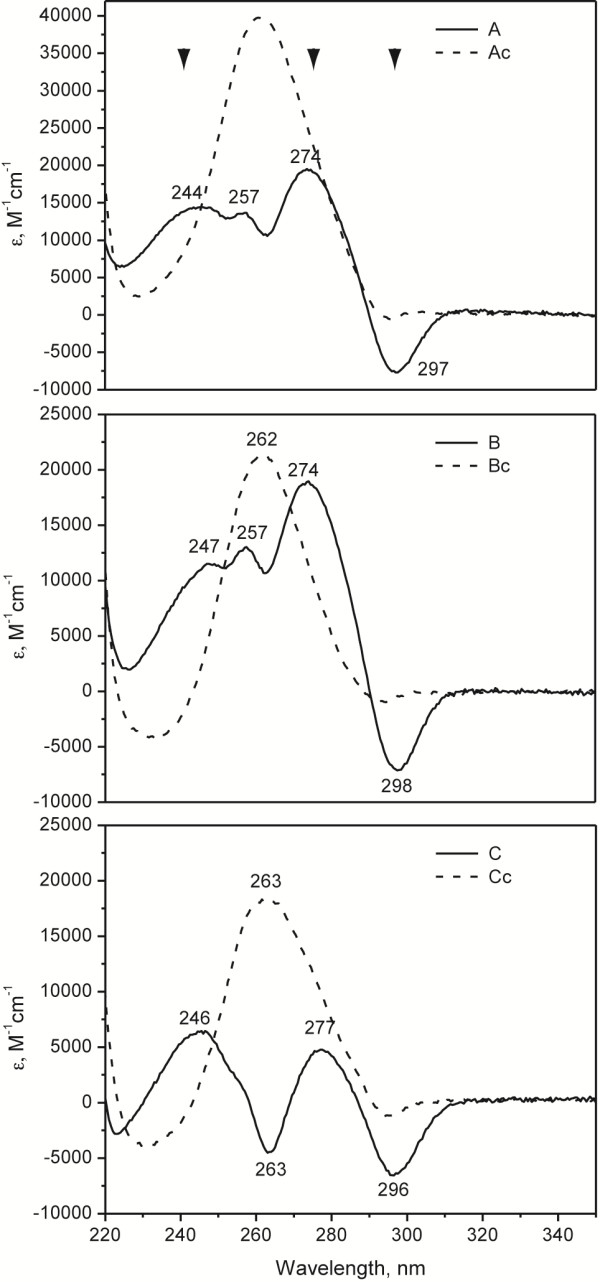
**UV–VIS thermal difference spectra (TDS) reveal quadruplex signatures for all tested mtDNA sequences with QFP.** GQ structure indicated via positive peaks at ~243 and ~273 nm and a negative peak at ~295 nm (indicated by arrowheads). The DNA sequences can be found in Table 2.

**Figure 6:**
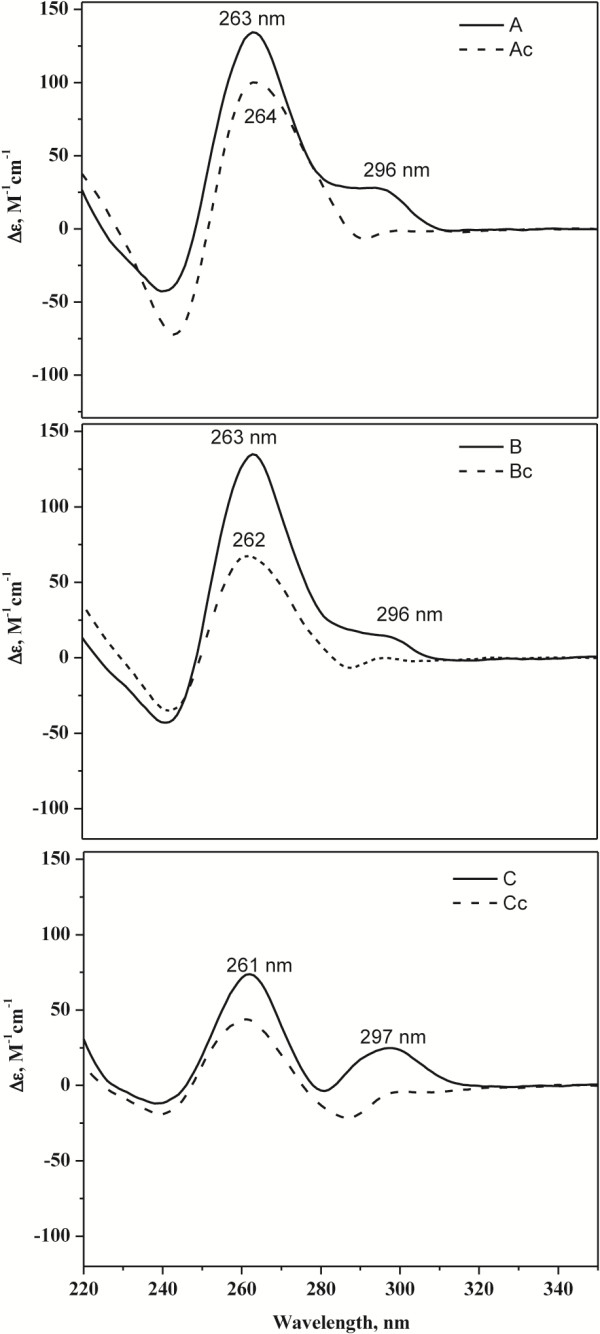
**CD wavelength scans for oligos A, B, and C and their corresponding control sequences.** CD spectroscopy can be used to differentiate among GQ topologies (e.g., a positive peak at ~264 nm and a negative peak at ~240 nm suggest a parallel component in GQ fold, whereas positive peaks at ~295 and ~245 nm and a negative peak at ~260 nm signify antiparallel component in GQ fold). Mixed-hybrid GQ structure have CD signature between those reported for parallel and antiparallel GQs. According to CD analysis, oligonucleotides containing A, B and C sequences have significant parallel character. In addition, C secondary structure has an antiparallel component.

**Figure 7:**
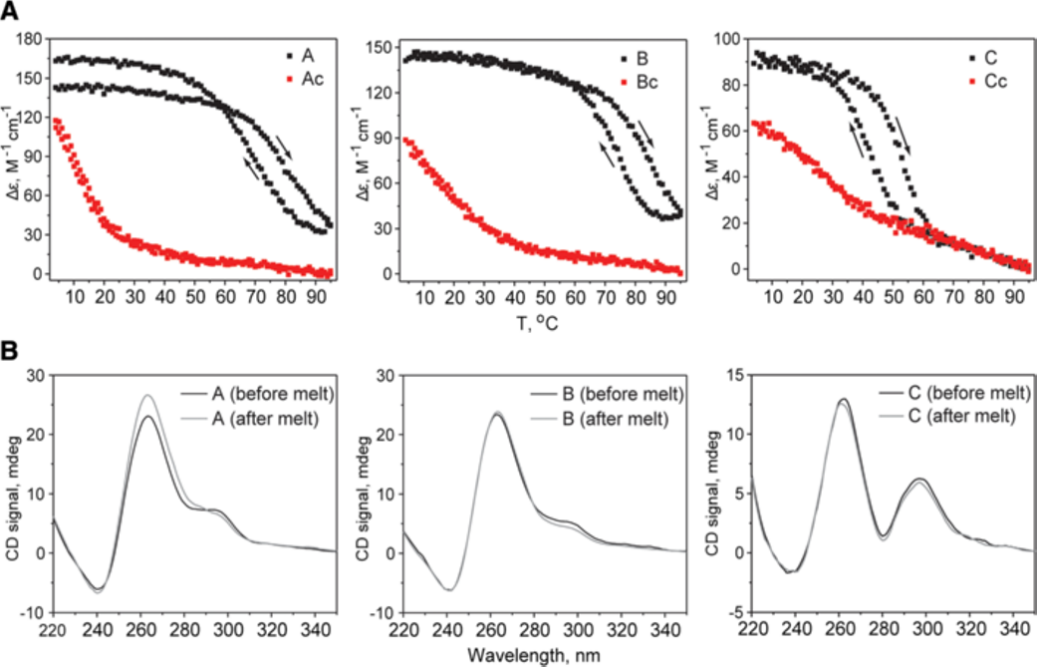
**Thermal stability studies for oligos A, B, and C and their control sequences. A)** DNA melting was monitored by CD at 264 nm in a 1 cm cell at 5 μM DNA concentration. Both melting and cooling curves are shown; arrows indicate the direction of temperature change. Note, hysteresis is observed for oligos A, B, and C but not for control sequences indicating that melting and cooling processes had differing intermediates. Temperature of the half-transition, T_1/2_, (and not the melting temperature, T_m_) was determined from melting curves only and is reported in Table 2. **B)** CD wavelength spectra were collected on each sample before and after melting. The CD signature of B and C oligos and all control samples did not change after melting thus indicating that each system returned to the original (most likely thermodynamic) secondary structure. CD signal at 264 nm for oligo A increased slightly after the melt signifying an increase in parallel folding after melt.

Because of the strong association of 2G QFP sequence motifs with mtDNA deletion breakpoints, we next performed standard biophysical analyses to determine whether representative 2G QFP sequences that overlap a mtDNA deletion breakpoint could form GQ structures *in vitro* (summarized in Table 2). The test oligonucleotides (A, B, and C) were compared to control sequences in which at least one guanine was changed to an adenine (Ac, Bc, and Cc). Using a combination of UV–VIS thermal difference spectra (TDS; Figure 5), UV region circular dichroism (CD) spectral signatures (Figure 6), and thermal stability tests (Figure 7), we show that A, B, and C oligos, but not their control counterparts, formed stable G-quadruplex secondary structures. For example, typical GQ structures can be identified via TDS by characteristic positive peaks at 243 and 273 nm, and a negative peak at 295 nm [47] (Figure 5 arrowheads). These peaks were observed in all three 2G QFP sequences but were missing in mutated control sequences. CD scans demonstrated that sequences A and B adopted a predominantly parallel GQ fold, while C demonstrated parallel folding with significant antiparallel component (Figure 6). Thermal melting/annealing of all secondary structures monitored by CD yielded high melting temperatures (T_1/2_) for A, B and C (Table 2) and showed that control sequences did not form stable secondary structures, as all of them melted below 27°C. The melting transitions for A, B, and C were characterized by a hysteresis of ~11°C, suggesting that their structural intermediates may differ during heating and cooling, yet at lower temperatures each sequence folded into a thermodynamically stable structure (Figure 7). The extent to which these oligos were forming intermolecular versus intramolecular GQ structures has not been addressed under these conditions, but either structure would be of potential biological significance. Our data clearly demonstrate the ability of mtDNA sequence to form *bona fide* G-quadruplexes in vitro. Additional experiments are needed to determine the full extent to which intra- and intermolecular GQs form *in vitro* and *in vivo*. Although our findings support a role for G-quadruplexes in breakpoint formation, we cannot rule out the possibility that intermolecular GQ motifs could also involved, or alternatively, compose elements of long imperfect repeats previously implicated in mtDNA deletion formation [44].

**Table 2:** **Summary of DNA biophysical characterization of representative 2G QFP and corresponding control sequences**

			Min. distance		Melting T _1/2_°C*
Oligo	Sequence	Midpoint	5′	3′	
A	GGA TGG GGT GGG GAG G	14813	27	0	80.0±1.5
Ac	**A**GA TGG **A**GT GG**A** GAG G				11.6±1.0
B	GGG GGA TGC GGG GG	13760	1	0	85.2±0.9
Bc	**A**GG **A**GA TGC **A**GG **A**G				14.0±2.0
C	GGA GGG TGG ATG G	5206	11	0	53.6±1.0
Cc	**A**GA GGG T**A**G ATG G				26.9±0.5

Oligonucleotides with QFP (A-C) or control sequences as indicted with (c) (mutations to ablate QFP are shown in bold) were tested for folding stability as reflected through their melting temperature, T_1/2_. Midpoints of these sequences, their minimum distance to a 5′ and 3′ breakpoint, and T_1/2_ are indicated. Melting temperatures were determined from CD melting curves as described in Methods section.

## Discussion

This study is the first to establish 2G QFP sequence prevalence in mammalian mtDNA, demonstrate their association with mtDNA breakpoints, and illustrate the capacity of 2G QFP mtDNA sequences to form quadruplex secondary structures. Because 3G QFP sequences have also been found within a few nucleotides of nuclear DNA deletion breakpoints [23], we suggest that both 2G and 3G GQ could contribute broadly to deletion formation in humans through a mechanism common to both nuclear and mitochondrial genomes.

Our work also significantly improves upon previous findings that 3G QFP are associated with mtDNA deletions [32]. The Oliveira study utilized a different QFP-determining protocol to identify five 3G QFP sequences (we report nine) and examined the relative enrichment of 5′ + 3′ pooled breakpoints identified from the Mitomap database ( http://www.mitomap.org) to these motif sequences. Two of the 3G QFP were located in the regions with prevalent 5′ or 3′ breakpoints and thus showed a high density of breakpoints within 100 bp, but not with random or partially randomized breakpoint sets. The authors concluded that these two 3G QFP were significantly associated with breakpoints; however, they did not exclude the effects of the non-random distribution of the breakpoints in their random controls. Our study improves upon these findings by expanding the 3G QFP sequences tested, using non-redundant separated 5′ and 3′ breakpoints, examining association from the motif and from the breakpoint, and using distribution matched controls.

One attractive potential mechanism for GQ contribution to deletion formation is that GQ structures contribute to mtDNA deletions during replication. Strong evidence supports replication-dependent deletion formation in the nucleus of cancer cells and yeast. The separation of DNA strands during replication is thought to leave the lagging strand vulnerable to GQ structure formation [19]. In yeast, when GQ structures are encountered by the replisome, DNA elongation is slowed [48]. The hallmarks of replication pausing were reversed by expression of PIF1 helicase, which has strong G-quadruplex resolving activity [49]. Repeat tandem arrays containing 3G QFP sequences are inherently unstable in yeast, but this sequence instability is prevented by expression of PIF1 [22]. The absence of PIF1, or exposure to G-quadruplex stabilizing compounds (ligands), slows replication and increases sequence instability at GQ repeat sequences [21]. Finally, the sites of nuclear genome instability in cancer cells frequently contain QFP, with a strong presence of 2G QFP sequences [23].

In mtDNA, the displaced heavy strand contains the majority of 2G QFP sequences (this study), which could be exposed by structural transitions induced by transcription, replication, or topological stress. It is worth noting that heavy strand DNA can be found at low levels as a single stranded DNA-RNA hybrid [50, 51] or bound by mtSSB [52], whose displacement or processing would allow exposed single-stranded DNA to fold into intramolecular GQ structures. RNA-DNA hybrids containing a GQ structure in the DNA have been suggested as mechanism of genome instability [53]. However they might arise, unresolved GQ structures in mtDNA may lead to collapsed replication forks, similar to those observed in the nucleus near stabilized GQ sequences [48]. If failed replication intermediates are repaired rather than degraded, the opportunity may arise to create recombinant mtDNA molecules [54]. We suggest that the increased density of predicted GQ structures in a region such as the boundaries of the common deletion or the deletion "hot spot" near the D-loop 3’ end [17, 18] (Figure 4) would increase the incidence of replication stalling, leading to the formation of single-stranded DNA or double-strand breaks, the latter of which are known to cause mtDNA deletion formation [55, 56] presumably through a non-homologous end joining (NHEJ) repair mechanism. SC structures have also been postulated to cause replication pausing, and have been associated with specific deletion breakpoints [18].

Circumstances that enhance the likelihood of replication fork collapse could increase mtDNA deletion formation rates. Besides structural interference by folded GQ DNA, nucleotide imbalances, defective polymerase, or oxidative damage could contribute to premature replication termination, the repair of which could lead to mtDNA deletions. Moreover, gene mutation or changes in expression levels during aging could also contribute to mtDNA deletion formation. Consistent with a role for replication in mtDNA deletion formation, the few known genes whose mutation is known to cause mtDNA deletion can be grouped into core replication subunits (POLG, POLG2, and Twinkle), nucleotide metabolism (TP and RRM2B), and nucleotide transport (ANT1, DNC, and potentially MPV17 [57]) (reviewed in [58, 59]). Altogether, these findings support replication pausing as a potential first step in mtDNA deletion formation.

Although the significant association of QFP sequences with breakpoints inspires the mechanisms proposed above, our results do not exclude others. The QFP sequences might also stimulate deletion formation during DNA repair through the formation of distant *trans* interactions. In addition, QFP might work in parallel with direct repeats or SC sequences, which have long been proposed to facilitate deletion formation during DNA repair [18, 54]. Interestingly, among all of the DNA motifs tested in this study, direct repeat is the only other sequence type significantly associated with breakpoints. To further demonstrate the significance of the association, we asked what the chances are that both ends of a deletion are in close vicinity to QFP or direct repeat sequences. Both p-values are small, p < 10^-14^ (QFP) and p < 10^-12^ (direct repeat) for the number of deletions with both ends within a 10 nt distance to the midpoint of the motifs (Additional file 9: Figure S6). For direct repeat sequences, we further determined the association of a deletion with a direct repeat pair, which decreases the p-value to <10^-14^ for the 10 nt distance. These findings strongly support the significant association of both QFP and direct repeat sequences with individual, or in the case of direct repeats, paired, deletion breakpoints.

GQ structures are surprisingly stable, and their resolution requires the action of specific proteins. In the nucleus, ATP-dependent helicases are dedicated to resolving these structures, and replication through QFP sequences is decreased in their absence [22, 48]. The presence of dedicated activities to manage the resolution of these structures suggests that GQ cause replication interference. In the nucleus, stabilization of GQ triggers the ataxia telangiectasia and Rad3-related protein (ATR) DNA damage response, consistent with single-stranded DNA formation at stalled replication forks [60] and exposure of single-stranded DNA gaps behind the replication fork [61]. As multiple different nuclear helicases are required to maintain nuclear genome stability and thus prevent disease, the maintenance of mtDNA stability likely requires multiple helicase activities in mitochondria, including Twinkle primase/helicase (PEO1) [62, 63], the 3′-5′ helicase RECQL4 [64], and the 5′-3′ helicase PIF1 [65–67]. Future studies describing interplay among replication, DNA repair, helicases, and GQ-forming sequences are likely to provide important insights into mitochondrial genome stability.

## Conclusion

In summary, our findings support the hypothesis that sequences predicted to form GQ structures in mtDNA are enriched with mtDNA deletion breakpoints. Among these multiple types of QFP, the 2G QFP motif set are in closest proximity to the largest number of breakpoints and overlap the most common breakpoints. The prediction that sequences of this motif set have the ability to form GQ was validated on a subset of sequences using *in vitro* structural analysis. Together, these findings suggest that GQ structures could be a major contributor to human mtDNA instability. Further experiments are needed to confirm these structures *in vivo* and establish their contribution to normal and pathological mitochondrial biology.

## Electronic supplementary material

Additional file 1: Figure S1: Diagram of heuristics used to generate the non-overlapping motif set. (PDF 101 KB)

Additional file 2: Table S1: Complete MDA tables for all motifs. (XLS 62 KB)

Additional file 3: Figure S2: Diagram of repeat pairs relative to mtDNA deletion breakpoint pairs and 2G QFP and 3G QFP sequences. Black lines connect the 5′ and 3′ breakpoints of 730 mtDNA deletions. On left, overlayed in orange lines are 5′ and 3′ locations of 11 nt direct repeat pairs and individual 2G QFP (green) and 3G QFP (gray) sequence positions. On right, overlayed in purple lines are 5′ and 3′ locations of inverted complementary repeat pairs and individual stemloop/cruciform (SC) structures (yellow-green). (PDF 2 MB)

Additional file 4: Table S2: Binomial p-values for mtDNA deletion breakpoints and 2G QFP, 3G QFP, and direct repeat sequence motifs. (XLS 16 KB)

Additional file 5: Figure S3: Histogram distribution of mtDNA deletions by clinical grouping. (PDF 618 KB)

Additional file 6: Table S3: 5′ or 3′ mtDNA deletion breakpoints from multiple clinical groupings show extensive association with 2G QFP and direct repeats. (PDF 163 KB)

Additional file 7: Figure S4: Histogram of 5′ and 3′ breakpoints in mtDNA deletions occuring in PSS, PEO, and PS. (PDF 314 KB)

Additional file 8: Figure S5: Venn diagram illustrating overlap among KSS, PEO, and PS deletions. (PDF 90 KB)

Additional file 9: Figure S6: Comparison of 2G QFP and direct repeat sequence enrichment with mtDNA deletions. Bar graph showing the number of deletions where both ends are within 10nt of 2G QFP sequence (left), of any individual direct repeat sequence (middle), or of direct repeat sequence pair (right) sequences. Control values are shown for reference with binomial p-values. (PDF 68 KB)
