# Supplementary material for: Association of G-quadruplex forming sequences with human mtDNA deletion breakpoints
Source: BMC Genomics. 2014 Aug 13;15(1):677. doi: 10.1186/1471-2164-15-677 (PMC4153896; doi:10.1186/1471-2164-15-677)
Supplement: Supplementary file 1 — Additional file 1: Figure S1: Diagram of heuristics used to generate the non-overlapping motif set. (PDF 101 KB) [file 12864_2014_6389_MOESM1_ESM.pdf]

1) Overlap with different QFP length,
select shorter one (x):

x GGTGGTAGGAGG
GGAACTGGTGGTAGG

2) Overlap with same QFP length but different number of G tetrads,
select the greater one (x):

x GGTTGGGGAATTTGTGGGGAGG
GGATCGGTTGGGGAATTTGTGG

3) Overlap with same QFP length and same number of G tetrads,
select both (x):

x GGAAAAGGAGGCTGG
x GGTTGGAAAAGGAGG

4) if no more overlap, use midpoint position, or
else, median of midpoints (diagrammed below):

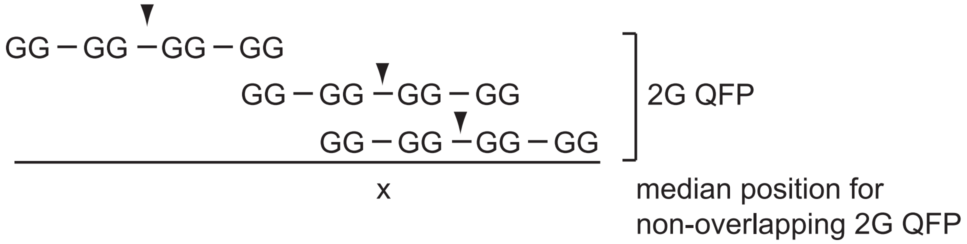

Additional file Figure S1: Diagram of heuristics used to generate the non-overlapping motif set.
