# Supplementary material for: Association of G-quadruplex forming sequences with human mtDNA deletion breakpoints
Source: BMC Genomics. 2014 Aug 13;15(1):677. doi: 10.1186/1471-2164-15-677 (PMC4153896; doi:10.1186/1471-2164-15-677)
Supplement: Supplementary file 3 — Additional file 3: Figure S2: Diagram of repeat pairs relative to mtDNA deletion breakpoint pairs and 2G QFP and 3G QFP sequences. Black lines connect the 5′ and 3′ breakpoints of 730 mtDNA deletions. On left, overlayed in orange lines are 5′ and 3′ locations of 11 nt direct repeat pairs and individual 2G QFP (green) and 3G QFP (gray) sequence positions. On right, overlayed in purple lines are 5′ and 3′ locations of inverted complementary repeat pairs and individual stemloop/cruciform (SC) structures (yellow-green). (PDF 2 MB) [file 12864_2014_6389_MOESM3_ESM.pdf]

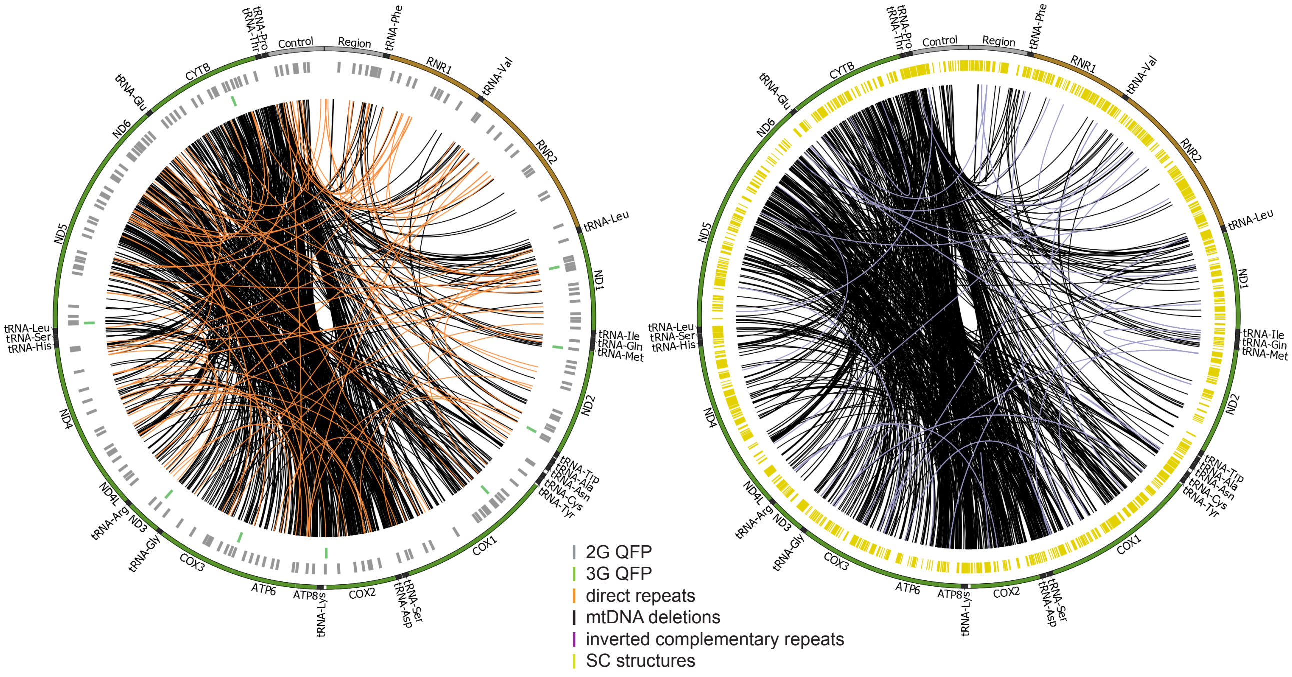

Additional file Figure S2. Diagram of repeat pairs relative to mtDNA deletion breakpoint pairs and 2G QFP and 3G QFP sequences. Black lines connect the 5’ and 3’ breakpoints of 730 mtDNA deletions. On left, overlayed in orange lines are 5’ and 3’ locations of 11 nt direct repeat pairs and individual 2G QFP (green) and 3G QFP (gray) sequence positions. On right, overlayed in purple lines are 5’ and 3’ locations of inverted complementary repeat pairs and individual stem-loop/cruciform (SC) structures (yellow-green).
