# Supplementary figures and images for: Association of G-quadruplex forming sequences with human mtDNA deletion breakpoints
Source: BMC Genomics. 2014 Aug 13;15(1):677. doi: 10.1186/1471-2164-15-677 (PMC4153896; doi:10.1186/1471-2164-15-677)

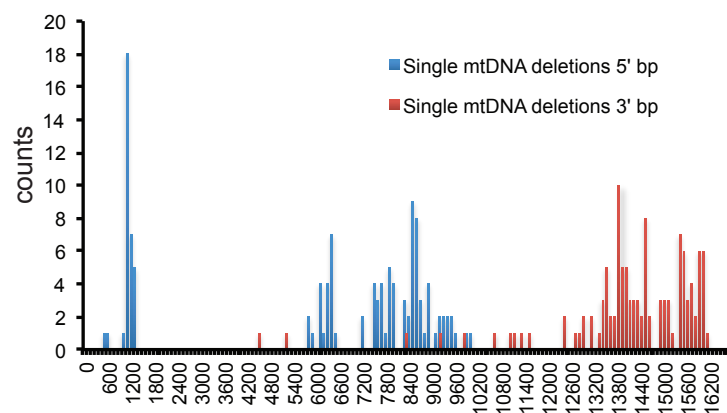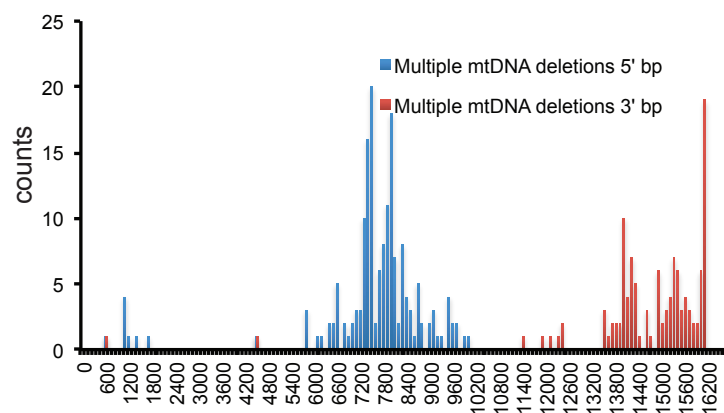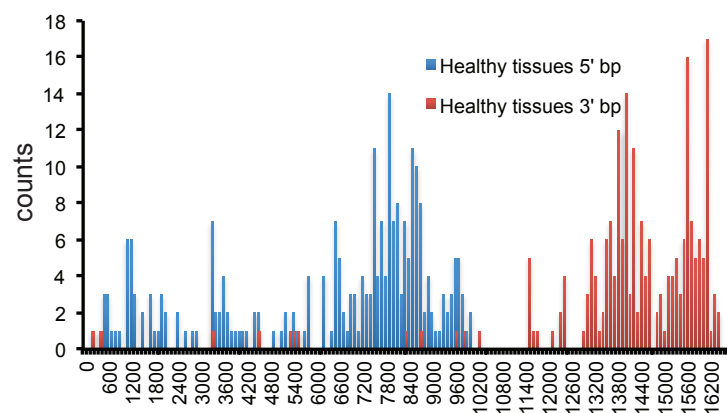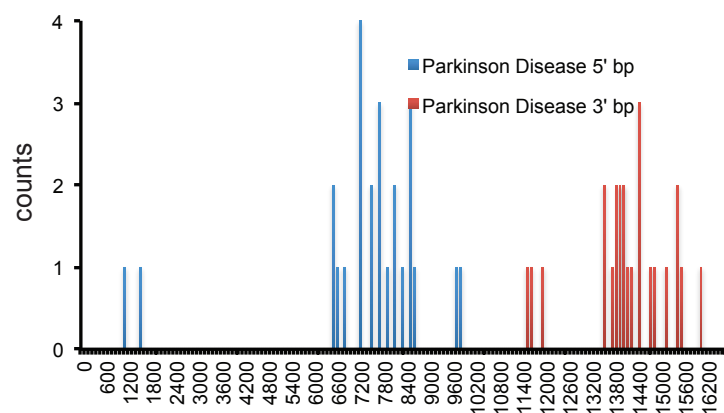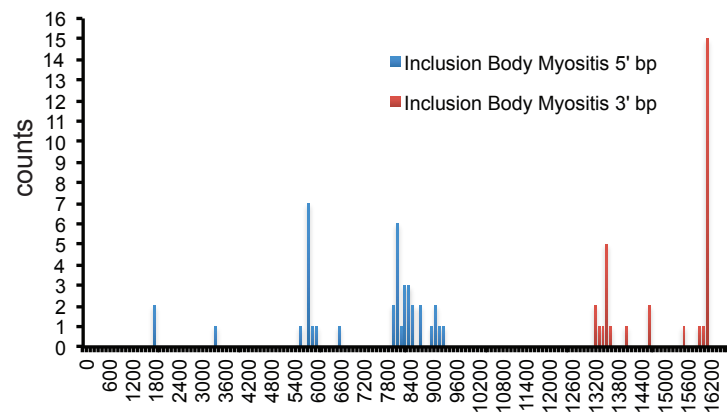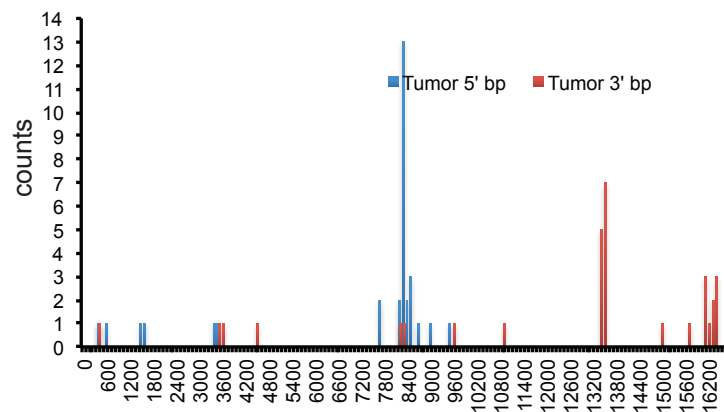

Additional file Figure S3. Histogram distribution of mtDNA deletions by clinical grouping.

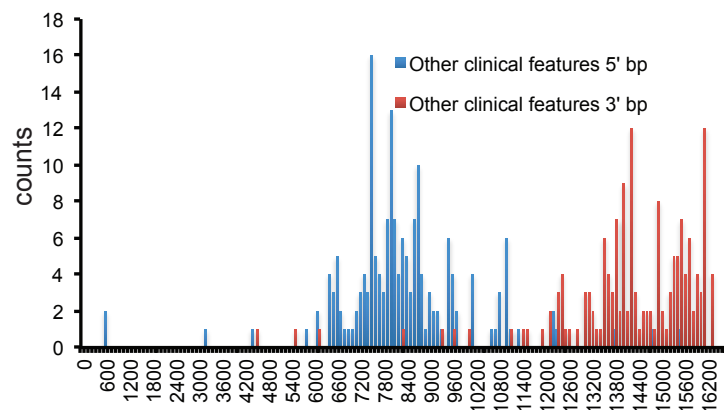

Supplement: Supplementary file 5 — Additional file 5: Figure S3: Histogram distribution of mtDNA deletions by clinical grouping. (PDF 618 KB) [file 12864_2014_6389_MOESM5_ESM.pdf]

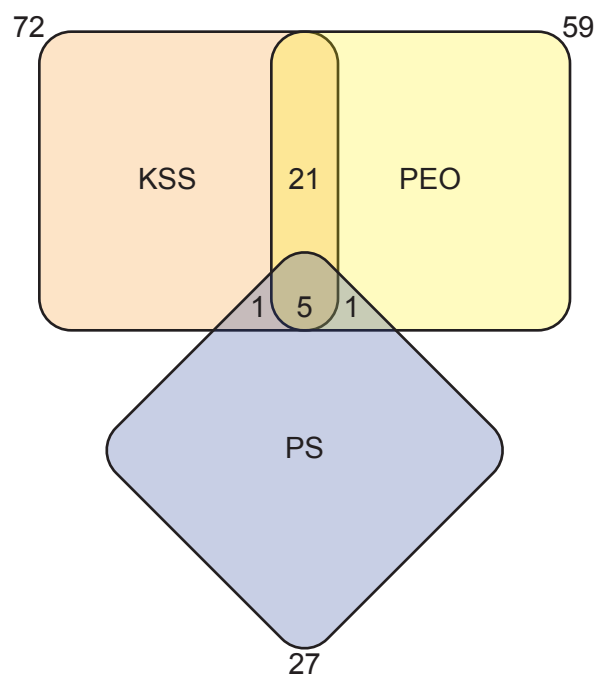

Additional file Figure S5. Venn diagram illustrating overlap among KSS, PEO, and PS deletions.

Supplement: Supplementary file 8 — Additional file 8: Figure S5: Venn diagram illustrating overlap among KSS, PEO, and PS deletions. (PDF 90 KB) [file 12864_2014_6389_MOESM8_ESM.pdf]
