# Supplementary material for: Association of G-quadruplex forming sequences with human mtDNA deletion breakpoints
Source: BMC Genomics. 2014 Aug 13;15(1):677. doi: 10.1186/1471-2164-15-677 (PMC4153896; doi:10.1186/1471-2164-15-677)
Supplement: Supplementary file 6 — Additional file 6: Table S3: 5′ or 3′ mtDNA deletion breakpoints from multiple clinical groupings show extensive association with 2G QFP and direct repeats. (PDF 163 KB) [file 12864_2014_6389_MOESM6_ESM.pdf]

Additional file Table S3. 5’ or 3’ mtDNA deletion breakpoints from multiple clinical groupings show extensive association with 2G QFP and direct repeats.

A. 5' breakpoint

	deletion #	5' b-p	b-p to motif: *3G ddi*	*3G QFP*	*2G ddi*	*2G QFP*	*direct rpts*	motif to b-p: *3G ddi*	*3G QFP*	*2G ddi*	*2G QFP*	*direct rpts*
healthy tissues	279	251	0.30	0.48	0.34	**0.0087**	0.10	**0.040**	0.32	0.18	0.81	0.89
inclusion body myositis	47	38	0.19	0.07	0.35	0.17	0.38	**0.014**	0.26	0.21	0.87	0.95
multiple mtDNA deletions	203	174	0.57	0.48	0.09	0.10	0.10	0.09	0.16	0.20	0.82	0.90
other clinical features	189	175	0.54	0.32	0.10	**0.0030**	**0.032**	0.15	**0.0077**	0.28	0.25	0.71
Parkinsons disease	25	25	0.51	0.54	0.73	0.10	0.26	0.09	0.38	0.32	0.86	0.93
single mtDNA deletions	127	118	0.36	0.81	0.63	0.17	0.051	**0.027**	0.32	0.28	0.82	0.90
tumor	32	31	0.45	0.19	0.69	0.48	0.30	0.06	0.38	0.26	0.74	0.82

p-values

B. 3' breakpoint

	deletion #	3' b-p	b-p to motif: *3G ddi*	*3G QFP*	*2G ddi*	*2G QFP*	*direct rpts*	motif to b-p: *3G ddi*	*3G QFP*	*2G ddi*	*2G QFP*	*direct rpts*
healthy tissues	279	215	0.80	0.64	0.32	**0.00048**	**0.017**	0.25	0.08	0.62	0.10	**0.008**
inclusion body myositis	47	31	0.74	0.61	0.28	0.13	0.16	0.97	0.92	0.94	0.12	0.051
multiple mtDNA deletions	203	116	0.76	0.60	0.14	**0.022**	**0.0018**	0.90	0.59	0.99	**0.019**	**0.034**
other clinical features	189	155	0.81	0.57	0.18	**0.0015**	**0.0016**	0.20	**0.0026**	0.33	**0.0010**	0.13
Parkinsons disease	25	24	0.85	0.82	0.31	**0.00030**	**0.005**	0.87	0.80	0.81	0.27	**0.0023**
single mtDNA deletions	127	117	0.81	0.69	**0.011**	**0.022**	0.09	0.56	0.08	0.57	0.052	0.09
tumor	32	31	0.79	0.59	**0.022**	**0.041**	0.23	0.55	0.48	0.46	0.12	0.19

p-values

C. 5' breakpoint

	deletion #	5' b-p	b-p to motif: *3G ddi*	*3G QFP*	*2G ddi*	*2G QFP*	*direct rpts*	motif to b-p: *3G ddi*	*3G QFP*	*2G ddi*	*2G QFP*	*direct rpts*
KSS	73	72	0.52	0.12	0.37	**0.00012**	**0.0040**	0.27	0.21	0.74	0.74	0.49
PEO	60	59	0.46	0.14	0.20	**0.0049**	**0.021**	0.27	0.23	0.65	0.75	0.50
PS	27	26	0.49	0.054	0.62	0.22	**0.0022**	0.28	**0.018**	0.37	0.73	0.59

p-values

D. 3' breakpoint

	deletion #	3' b-p	b-p to motif: *3G ddi*	*3G QFP*	*2G ddi*	*2G QFP*	*direct rpts*	motif to b-p: *3G ddi*	*3G QFP*	*2G ddi*	*2G QFP*	*direct rpts*
KSS	73	69	0.73	0.48	0.07	**0.031**	0.13	0.056	0.32	0.55	0.07	0.09
PEO	60	57	0.74	0.07	**0.0021**	**0.011**	0.13	0.28	0.26	0.64	**0.0010**	0.08
PS	27	26	0.66	0.48	0.12	0.17	**0.045**	0.68	0.16	0.32	0.10	0.17

p-values

A) 5’ breakpoint minimum distance analysis for 3G and 2G (QFP and ddi) from clinically categorized mtDNA deletions. B) 3’ breakpoint minimum distance analysis for 3G and 2G (QFP and ddi) from clinically categorized mtDNA deletions. C) 5’ breakpoints from KSS, PEO, or PS diseases subdivided from single mtDNA deletions group. D) 3’ breakpoints from KSS, PEO, or PS diseases subdivided from single mtDNA deletions group. Two significant digits are shown for p-values with statistical significance (<0.05, shown in bold).
