# Supplementary material for: Association of G-quadruplex forming sequences with human mtDNA deletion breakpoints
Source: BMC Genomics. 2014 Aug 13;15(1):677. doi: 10.1186/1471-2164-15-677 (PMC4153896; doi:10.1186/1471-2164-15-677)
Supplement: Supplementary file 7 — Additional file 7: Figure S4: Histogram of 5′ and 3′ breakpoints in mtDNA deletions occuring in PSS, PEO, and PS. (PDF 314 KB) [file 12864_2014_6389_MOESM7_ESM.pdf]

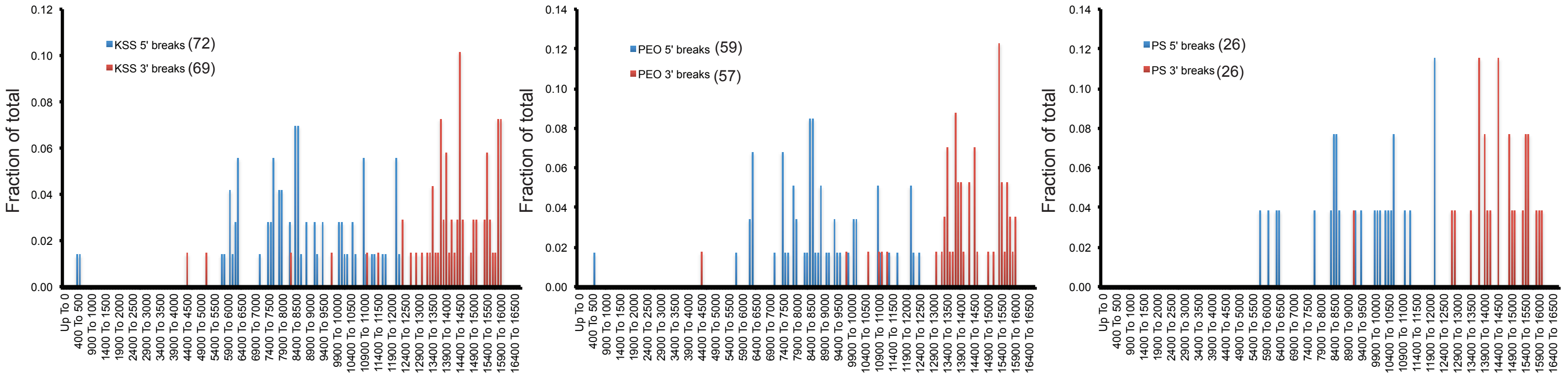

KSS: 73 deletions PEO: 60 deletions PS: 27 deletions

Additional file Figure S4. Histogram of 5’ and 3‘ breakpoints in mtDNA deletions occuring in PSS, PEO, and PS.
