# Supplementary material for: Association of G-quadruplex forming sequences with human mtDNA deletion breakpoints
Source: BMC Genomics. 2014 Aug 13;15(1):677. doi: 10.1186/1471-2164-15-677 (PMC4153896; doi:10.1186/1471-2164-15-677)
Supplement: Supplementary file 9 — Additional file 9: Figure S6: Comparison of 2G QFP and direct repeat sequence enrichment with mtDNA deletions. Bar graph showing the number of deletions where both ends are within 10nt of 2G QFP sequence (left), of any individual direct repeat sequence (middle), or of direct repeat sequence pair (right) sequences. Control values are shown for reference with binomial p-values. (PDF 68 KB) [file 12864_2014_6389_MOESM9_ESM.pdf]

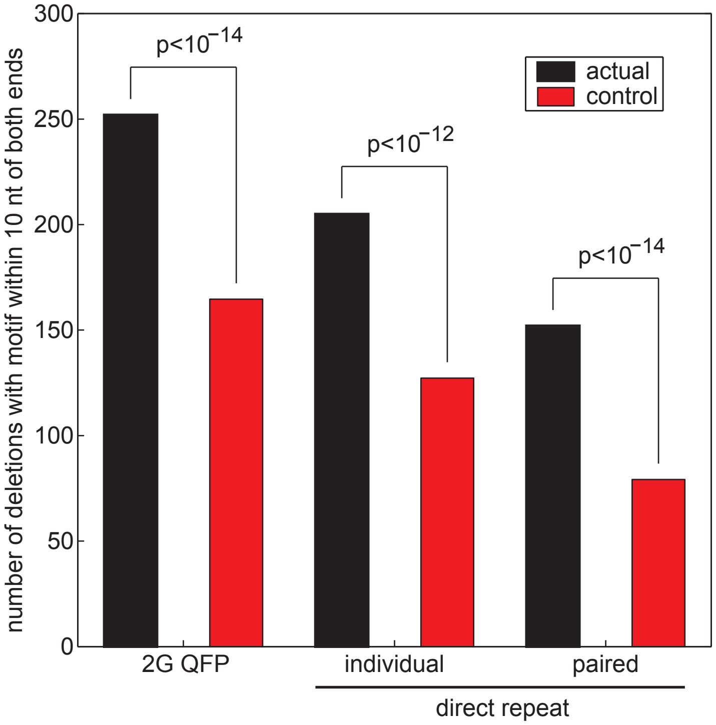

Additional file Figure S6. Comparison of 2G QFP and direct repeat sequence enrichment with mtDNA deletions. Bar graph showing the number of deletions where both ends are within 10nt of 2G QFP sequence (left), of any individual direct repeat sequence (middle), or of direct repeat sequence pair (right) sequences. Control values are shown for reference with binomial p-values.
